# Supplementary material for: MetaScore: A Novel Machine-Learning-Based Approach to Improve Traditional Scoring Functions for Scoring Protein–Protein Docking Conformations
Source: Biomolecules. 2023 Jan 6;13(1):121. doi: 10.3390/biom13010121 (PMC9855734; doi:10.3390/biom13010121)
Supplement: Supplementary file 1 [file biomolecules-13-00121-s001.zip › biomolecules-2102881-supplementary.pdf]

Supplementary Materials

MetaScore: A Novel Machine-Learning-Based Approach to Improve Traditional Scoring Functions for Scoring Protein–Protein Docking Conformations

Yong Jung [1,2,3], Cunliang Geng [4], Alexandre M. J. J. Bonvin [4], Li C. Xue [4,5,*] and Vasant G. Honavar [1,2,3,6,7,8,9,*]

[1] Bioinformatics & Genomics Graduate Program, Pennsylvania State University, University Park, PA 16802, USA; yong.joung@gmail.com
[2] Artificial Intelligence Research Laboratory, Pennsylvania State University, University Park, PA 16802, USA
[3] Huck Institutes of the Life Sciences, Pennsylvania State University, University Park, PA 16802, USA
[4] Bijvoet Centre for Biomolecular Research, Faculty of Science—Chemistry, Utrecht University, Padualaan 8, 3584 CH Utrecht, The Netherlands; gengcunliang@gmail.com (C.G.); a.m.j.j.bonvin@uu.nl (A.M.J.J.B.)
[5] Center for Molecular and Biomolecular Informatics, Radboudumc, Greet Grooteplein 26-28, 6525 GA Nijmegen, The Netherlands
[6] Clinical and Translational Sciences Institute, Pennsylvania State University, University Park, PA 16802, USA
[7] College of Information Sciences & Technology, Pennsylvania State University, University Park, PA 16802, USA
[8] Institute for Computational and Data Sciences, Pennsylvania State University, University Park, PA 16802, USA
[9] Center for Big Data Analytics and Discovery Informatics, Pennsylvania State University, University Park, PA 16823, USA
* Correspondence: li.xue@radboudumc.nl (L.C.X.); vhonavar@psu.edu (V.G.H.); Tel.: +31-61-859-4390 (L.C.X.); +1-814-865-3141 (V.G.H.)

Supplementary Table S1. BLAST parameters.

Query Length	Substitution Matrix	Gap Open Cost	Gap Extend Cost
<35	PAM-30	9	1
35-50	PAM-70	10	1
50-85	BLOSUM-80	10	1
>85	BLOSUM-62	11	1

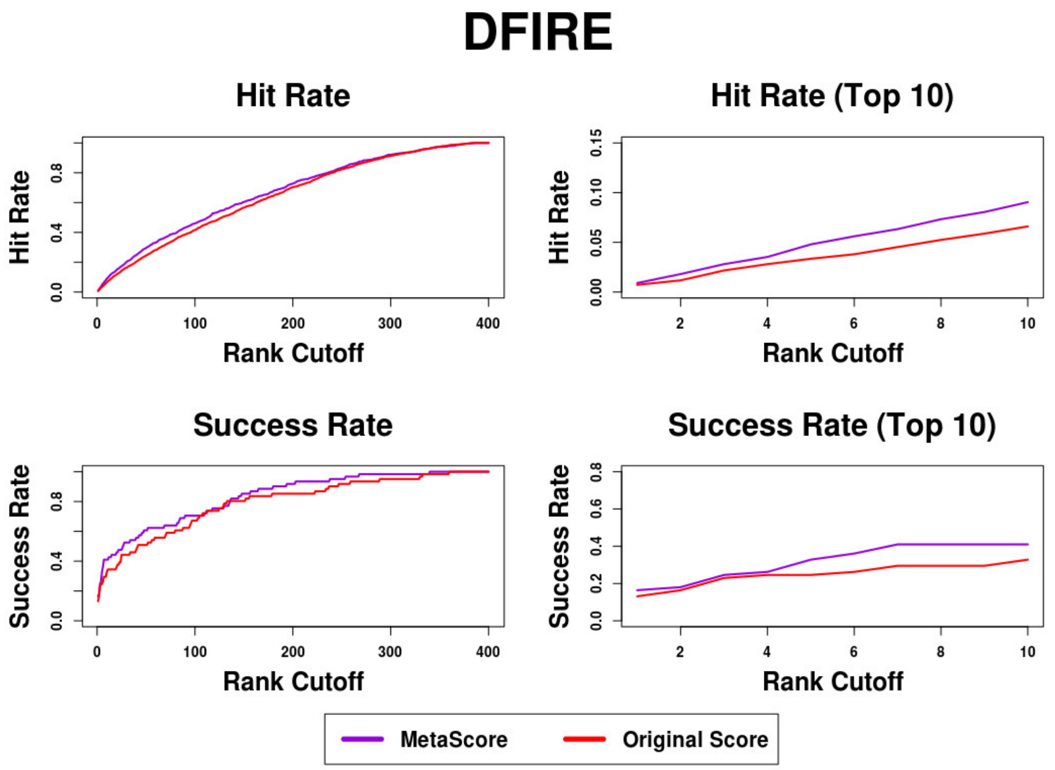

Supplementary Figure S1. Success rates and hit rates plotted against the top m conformations for a classical scoring method (DFIRE), machine learning-based method using RF, and the combined method of the two methods using BM4 decoy set. There are four panels. Top-left panel shows hit rates for conformations of top m ranging from 1 to 400; Top-right panel shows hit rates for conformations of top m ranging from 1 to 10; Bottom-left panel shows success rates for conformations of top m ranging from 1 to 400; Bottom-right panel shows success rates for conformations of top m ranging from 1 to 10.

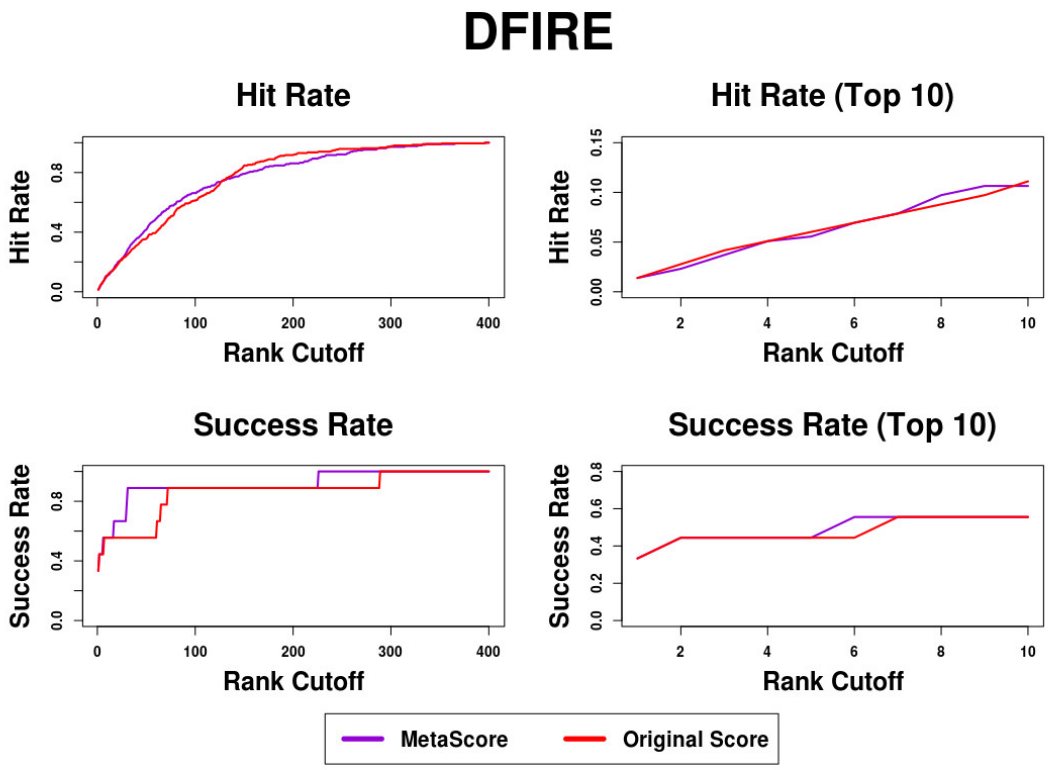

Supplementary Figure S2. Success rates and hit rates plotted against the top m conformations for a classical scoring method (DFIRE), machine learning-based method using RF, and the combined method of the two methods using BM5 decoy set. There are four panels. Top-left panel shows hit rates for conformations of top m ranging from 1 to 400; Top-right panel shows hit rates for conformations of top m ranging from 1 to 10; Bottom-left panel shows success rates for conformations of top m ranging from 1 to 400; Bottom-right panel shows success rates for conformations of top m ranging from 1 to 10.

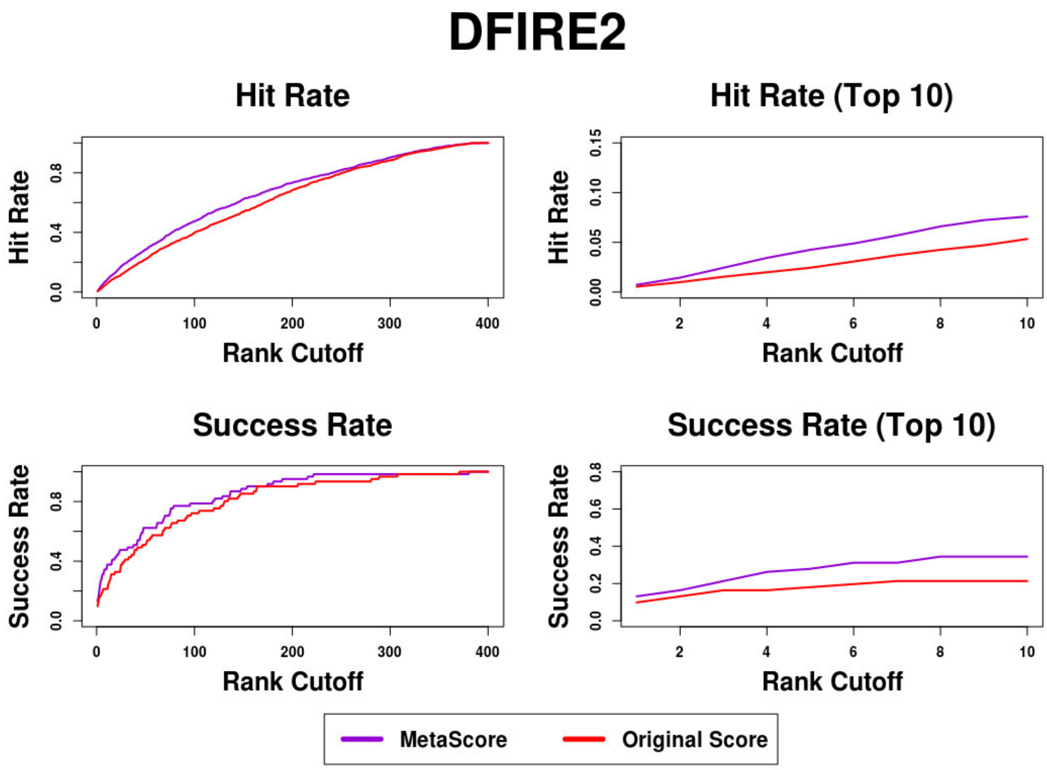

Supplementary Figure S3. Success rates and hit rates plotted against the top m conformations for a classical scoring method (DFIRE2), machine learning-based method using RF, and the combined method of the two methods using BM4 decoy set. There are four panels. Top-left panel shows hit rates for conformations of top m ranging from 1 to 400; Top-right panel shows hit rates for conformations of top m ranging from 1 to 10; Bottom-left panel shows success rates for conformations of top m ranging from 1 to 400; Bottom-right panel shows success rates for conformations of top m ranging from 1 to 10.

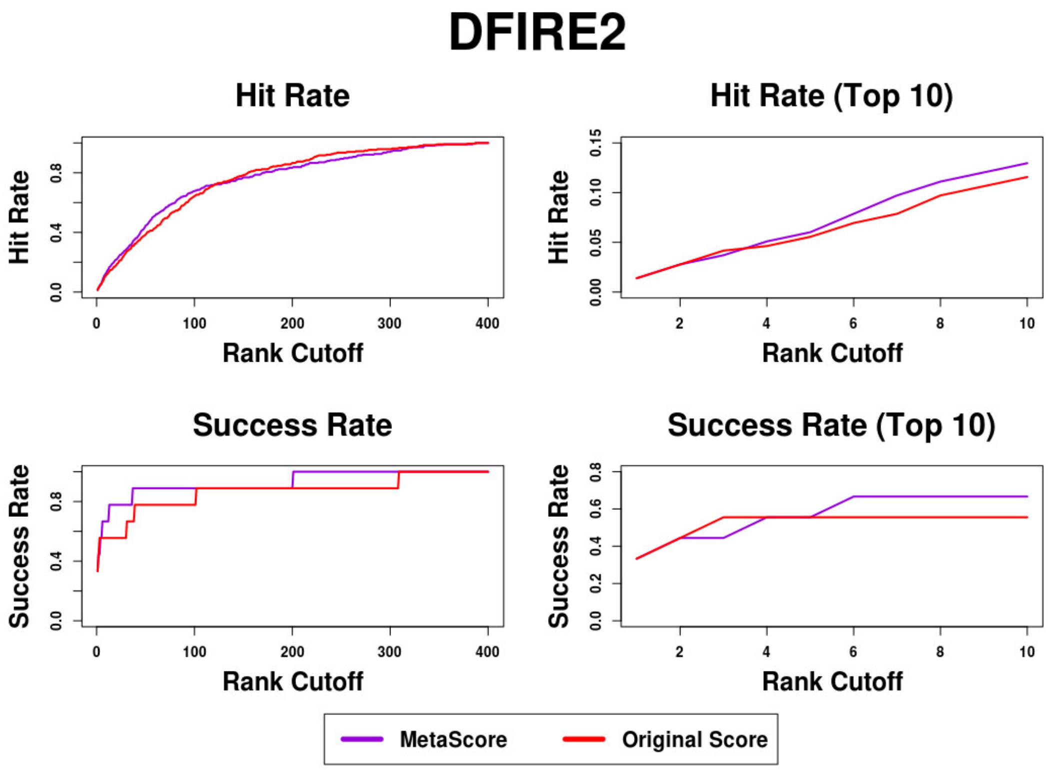

Supplementary Figure S4. Success rates and hit rates plotted against the top m conformations for a classical scoring method (DFIRE2), machine learning-based method using RF, and the combined method of the two methods using BM5 decoy set. There are four panels. Top-left panel shows hit rates for conformations of top m ranging from 1 to 400; Top-right panel shows hit rates for conformations of top m ranging from 1 to 10; Bottom-left panel shows success rates for conformations of top m ranging from 1 to 400; Bottom-right panel shows success rates for conformations of top m ranging from 1 to 10.

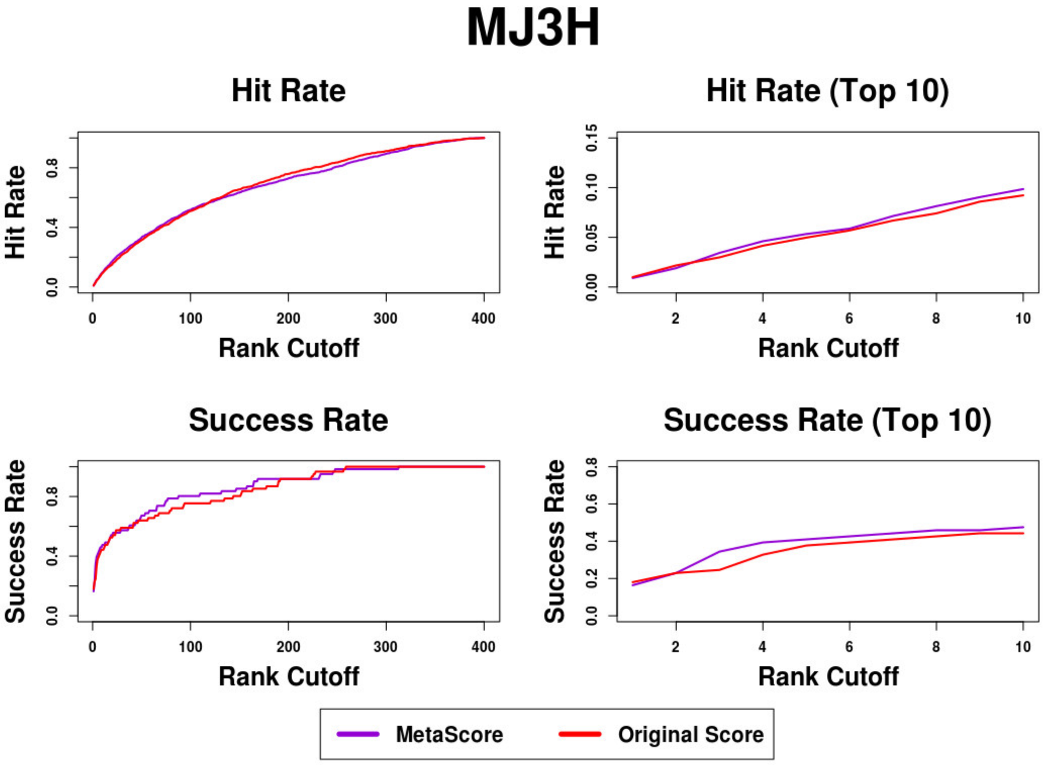

Supplementary Figure S5. Success rates and hit rates plotted against the top m conformations for a classical scoring method (MJ3H), machine learning-based method using RF, and the combined method of the two methods using BM4 decoy set. There are four panels. Top-left panel shows hit rates for conformations of top m ranging from 1 to 400; Top-right panel shows hit rates for conformations of top m ranging from 1 to 10; Bottom-left panel shows success rates for conformations of top m ranging from 1 to 400; Bottom-right panel shows success rates for conformations of top m ranging from 1 to 10.

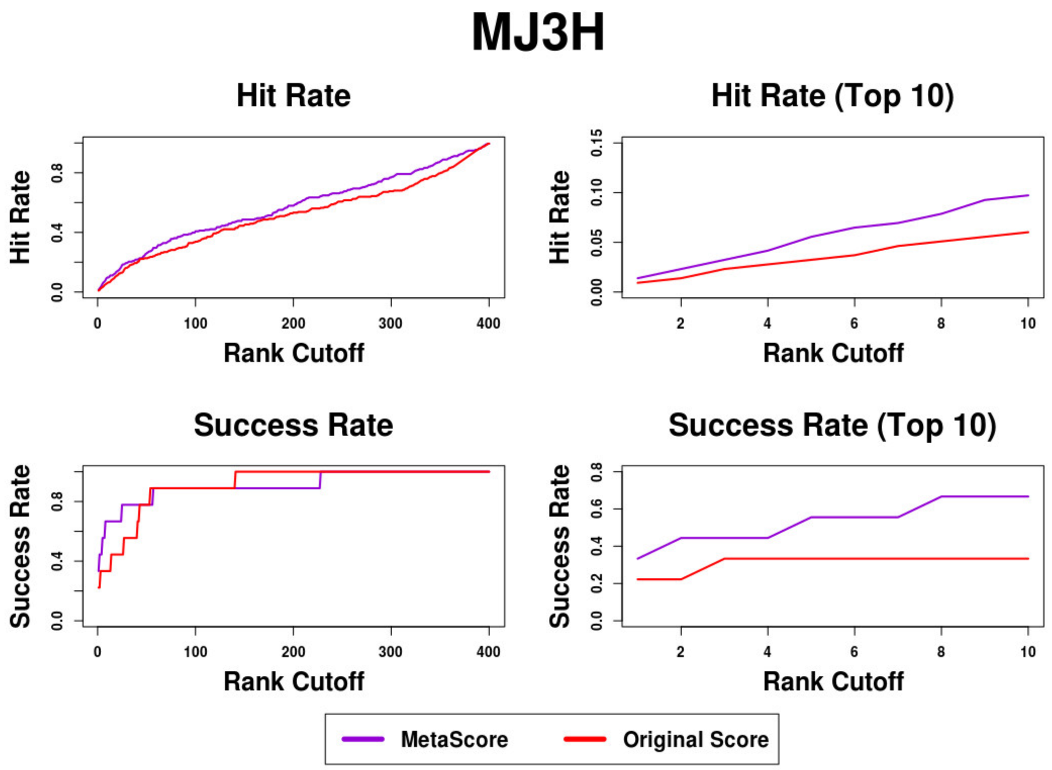

Supplementary Figure S6. Success rates and hit rates plotted against the top m conformations for a classical scoring method (MJ3H), machine learning-based method using RF, and the combined method of the two methods using BM5 decoy set. There are four panels. Top-left panel shows hit rates for conformations of top m ranging from 1 to 400; Top-right panel shows hit rates for conformations of top m ranging from 1 to 10; Bottom-left panel shows success rates for conformations of top m ranging from 1 to 400; Bottom-right panel shows success rates for conformations of top m ranging from 1 to 10.

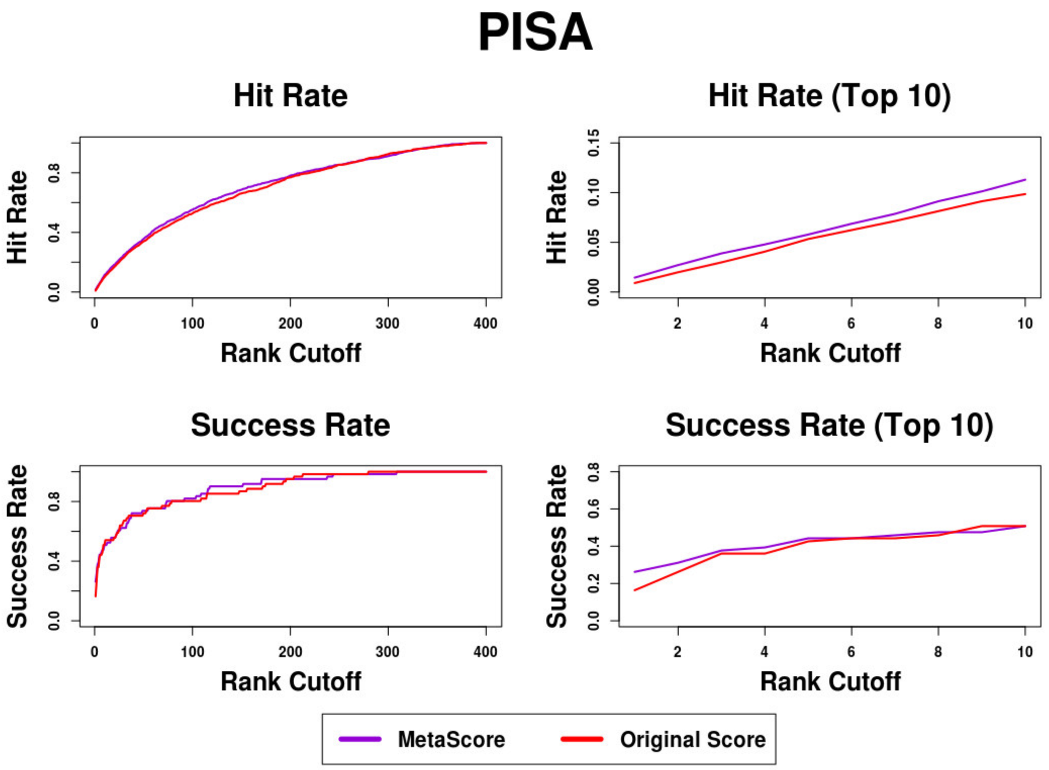

Supplementary Figure S7. Success rates and hit rates plotted against the top m conformations for a classical scoring method (PISA), machine learning-based method using RF, and the combined method of the two methods using BM4 decoy set. There are four panels. Top-left panel shows hit rates for conformations of top m ranging from 1 to 400; Top-right panel shows hit rates for conformations of top m ranging from 1 to 10; Bottom-left panel shows success rates for conformations of top m ranging from 1 to 400; Bottom-right panel shows success rates for conformations of top m ranging from 1 to 10.

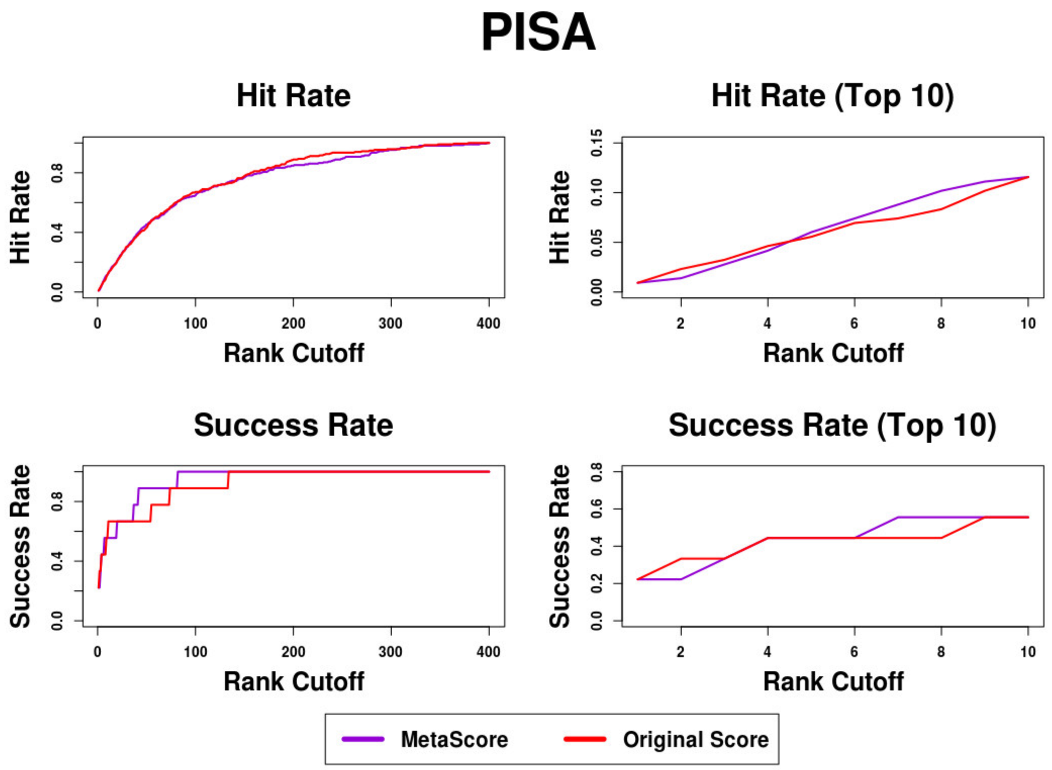

Supplementary Figure S8. Success rates and hit rates plotted against the top m conformations for a classical scoring method (PISA), machine learning-based method using RF, and the combined method of the two methods using BM5 decoy set. There are four panels. Top-left panel shows hit rates for conformations of top m ranging from 1 to 400; Top-right panel shows hit rates for conformations of top m ranging from 1 to 10; Bottom-left panel shows success rates for conformations of top m ranging from 1 to 400; Bottom-right panel shows success rates for conformations of top m ranging from 1 to 10.

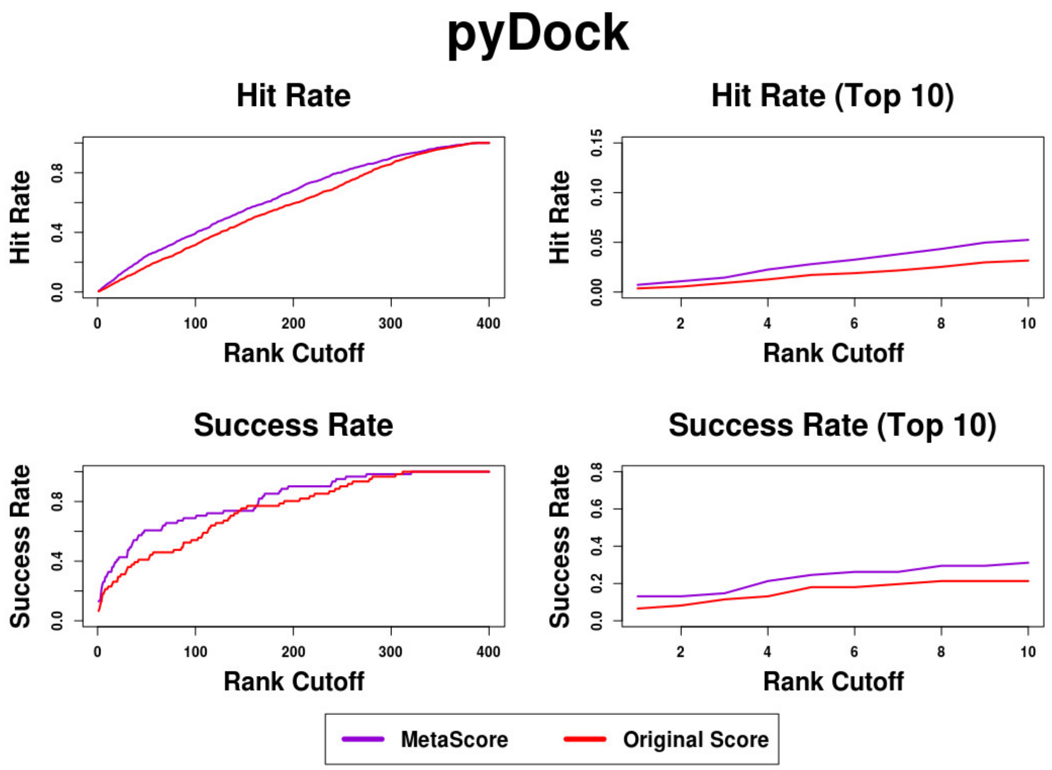

Supplementary Figure S9. Success rates and hit rates plotted against the top m conformations for a classical scoring method (pyDock), machine learning-based method using RF, and the combined method of the two methods using BM4 decoy set. There are four panels. Top-left panel shows hit rates for conformations of top m ranging from 1 to 400; Top-right panel shows hit rates for conformations of top m ranging from 1 to 10; Bottom-left panel shows success rates for conformations of top m ranging from 1 to 400; Bottom-right panel shows success rates for conformations of top m ranging from 1 to 10.

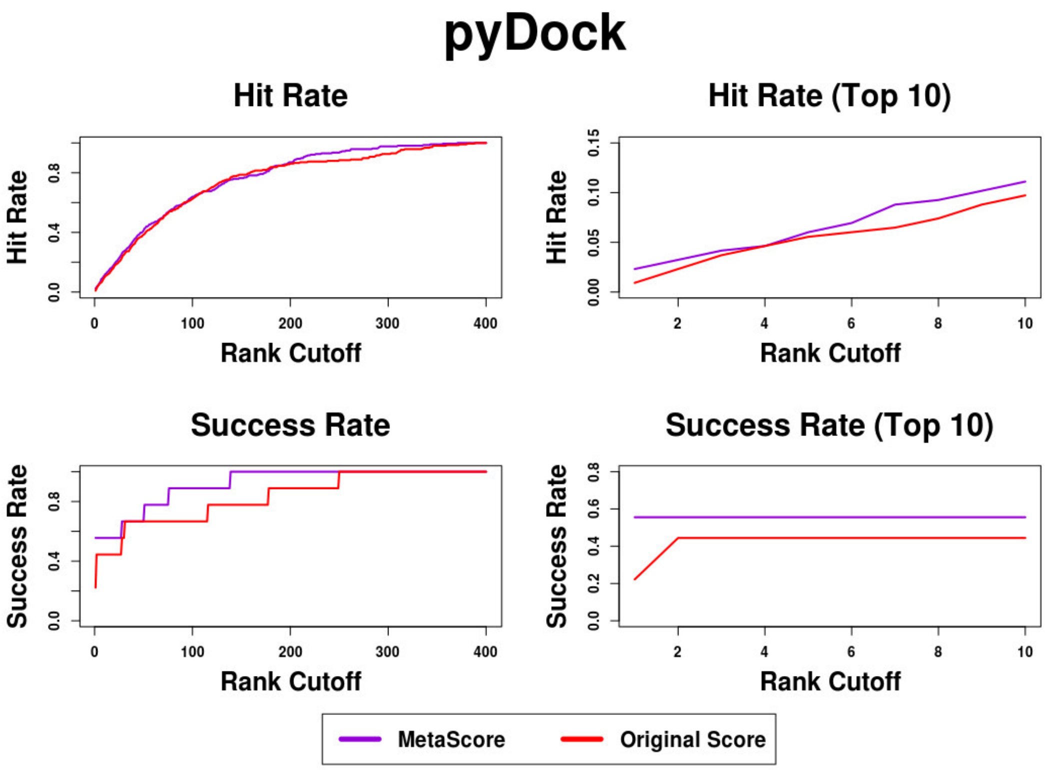

Supplementary Figure S10. Success rates and hit rates plotted against the top m conformations for a classical scoring method (pyDock), machine learning-based method using RF, and the combined method of the two methods using BM5 decoy set. There are four panels. Top-left panel shows hit rates for conformations of top m ranging from 1 to 400; Top-right panel shows hit rates for conformations of top m ranging from 1 to 10; Bottom-left panel shows success rates for conformations of top m ranging from 1 to 400; Bottom-right panel shows success rates for conformations of top m ranging from 1 to 10.

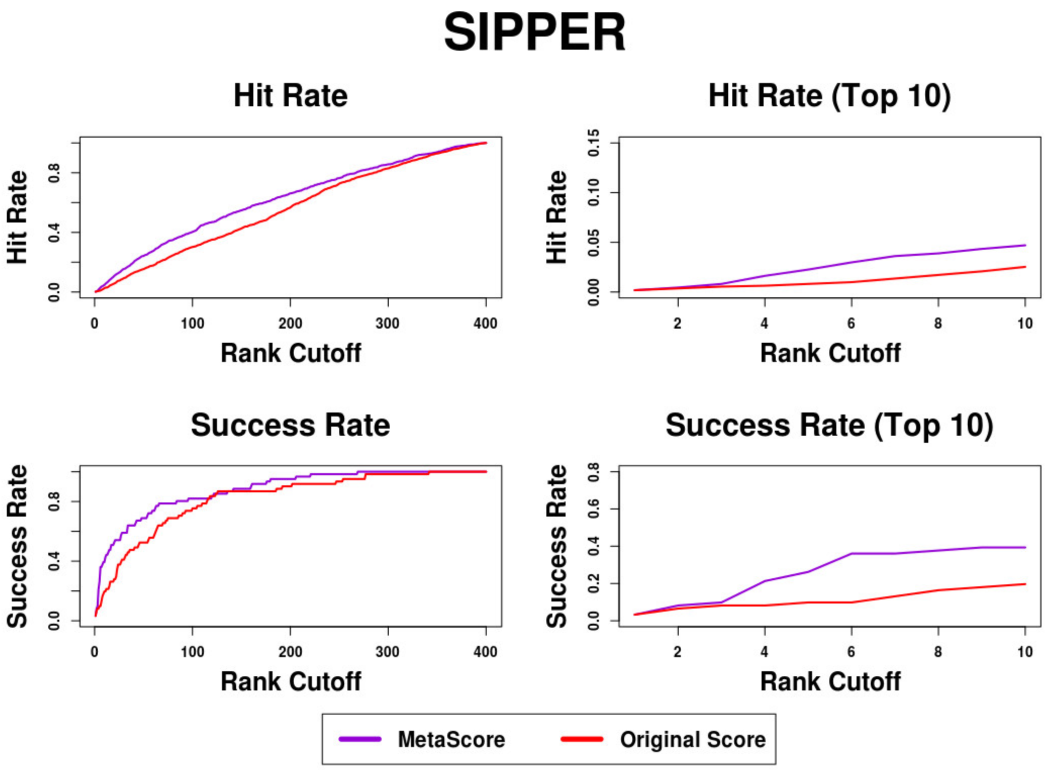

Supplementary Figure S11. Success rates and hit rates plotted against the top m conformations for a classical scoring method (SIPPER), machine learning-based method using RF, and the combined method of the two methods using BM4 decoy set. There are four panels. Top-left panel shows hit rates for conformations of top m ranging from 1 to 400; Top-right panel shows hit rates for conformations of top m ranging from 1 to 10; Bottom-left panel shows success rates for conformations of top m ranging from 1 to 400; Bottom-right panel shows success rates for conformations of top m ranging from 1 to 10.

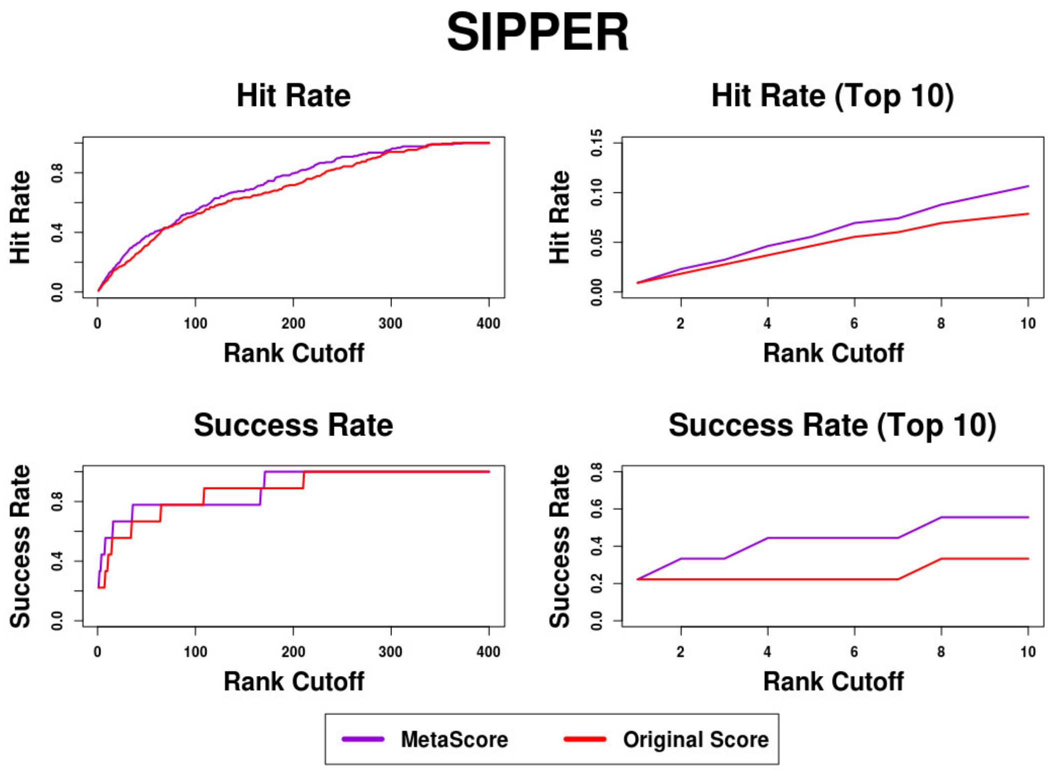

Supplementary Figure S12. Success rates and hit rates plotted against the top m conformations for a classical scoring method (SIPPER), machine learning-based method using RF, and the combined method of the two methods using BM5 decoy set. There are four panels. Top-left panel shows hit rates for conformations of top m ranging from 1 to 400; Top-right panel shows hit rates for conformations of top m ranging from 1 to 10; Bottom-left panel shows success rates for conformations of top m ranging from 1 to 400; Bottom-right panel shows success rates for conformations of top m ranging from 1 to 10.

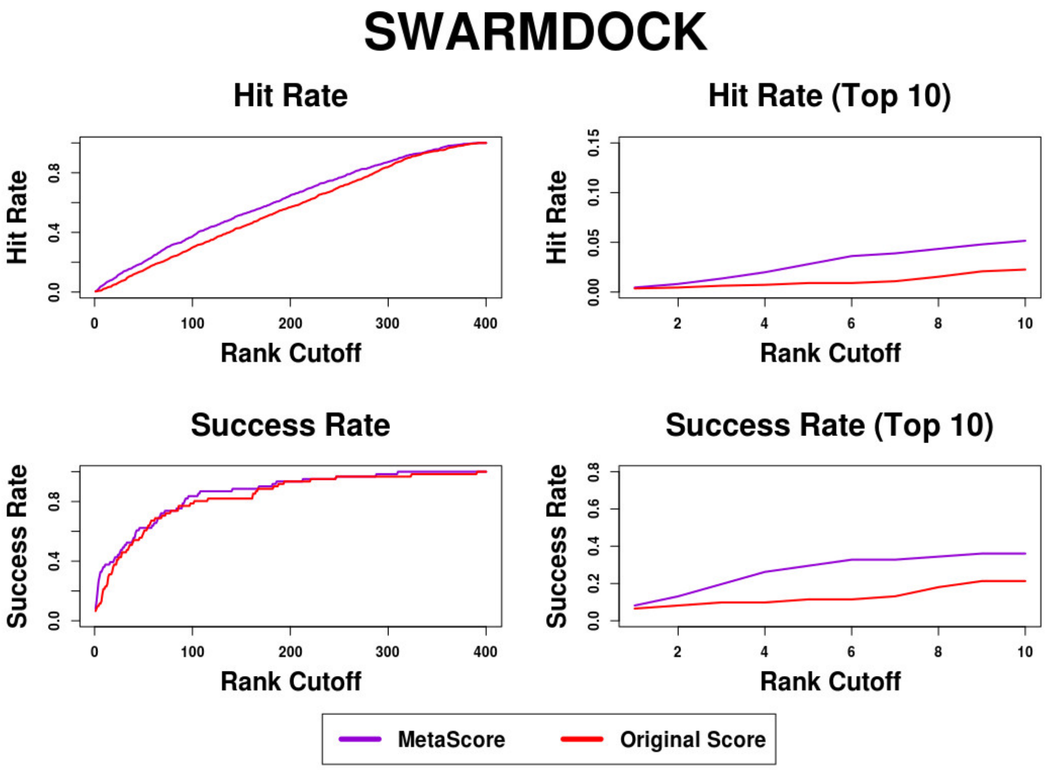

Supplementary Figure S13. Success rates and hit rates plotted against the top m conformations for a classical scoring method (SWARMDOCK), machine learning-based method using RF, and the combined method of the two methods using BM4 decoy set. There are four panels. Top-left panel shows hit rates for conformations of top m ranging from 1 to 400; Top-right panel shows hit rates for conformations of top m ranging from 1 to 10; Bottom-left panel shows success rates for conformations of top m ranging from 1 to 400; Bottom-right panel shows success rates for conformations of top m ranging from 1 to 10.

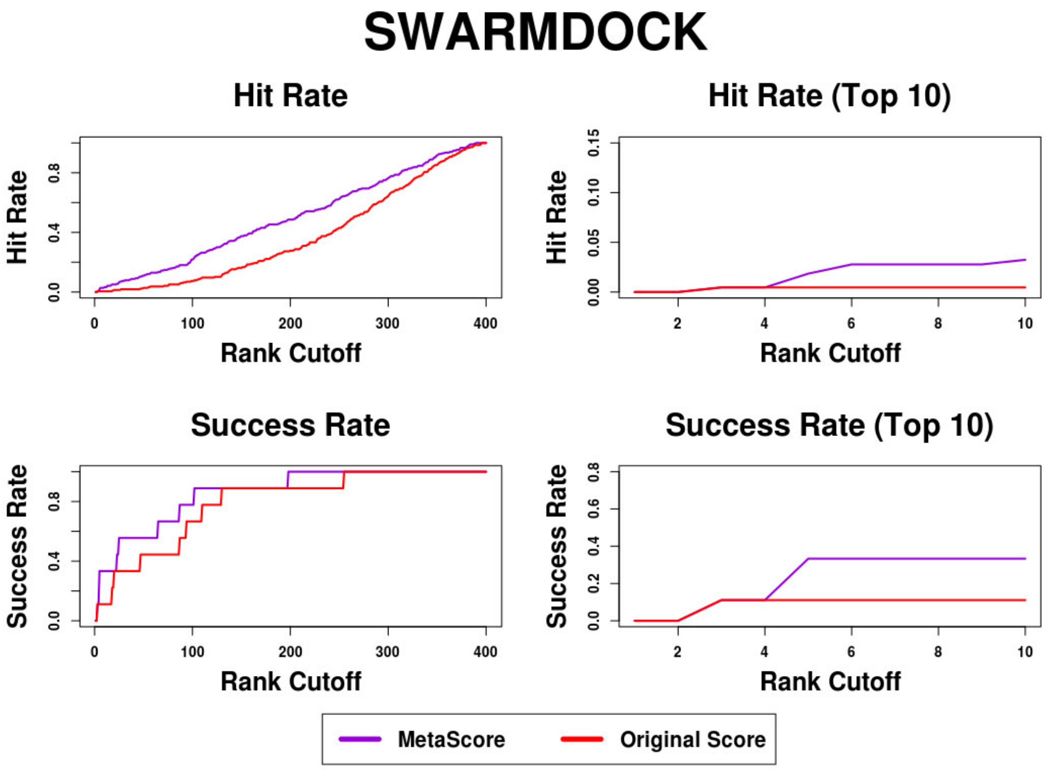

Supplementary Figure S14. Success rates and hit rates plotted against the top m conformations for a classical scoring method (SWARMDOCK), machine learning-based method using RF, and the combined method of the two methods using BM5 decoy set. There are four panels. Top-left panel shows hit rates for conformations of top m ranging from 1 to 400; Top-right panel shows hit rates for conformations of top m ranging from 1 to 10; Bottom-left panel shows success rates for conformations of top m ranging from 1 to 400; Bottom-right panel shows success rates for conformations of top m ranging from 1 to 10.

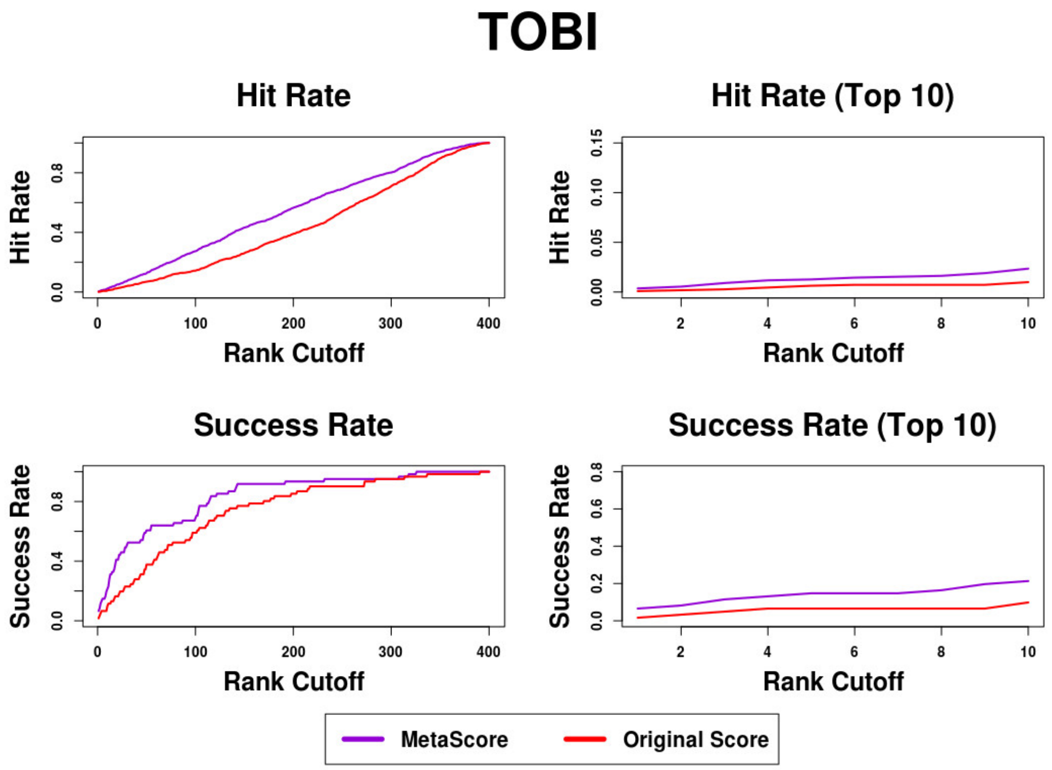

Supplementary Figure S15. Success rates and hit rates plotted against the top m conformations for a classical scoring method (TOBI), machine learning-based method using RF, and the combined method of the two methods using BM4 decoy set. There are four panels. Top-left panel shows hit rates for conformations of top m ranging from 1 to 400; Top-right panel shows hit rates for conformations of top m ranging from 1 to 10; Bottom-left panel shows success rates for conformations of top m ranging from 1 to 400; Bottom-right panel shows success rates for conformations of top m ranging from 1 to 10.

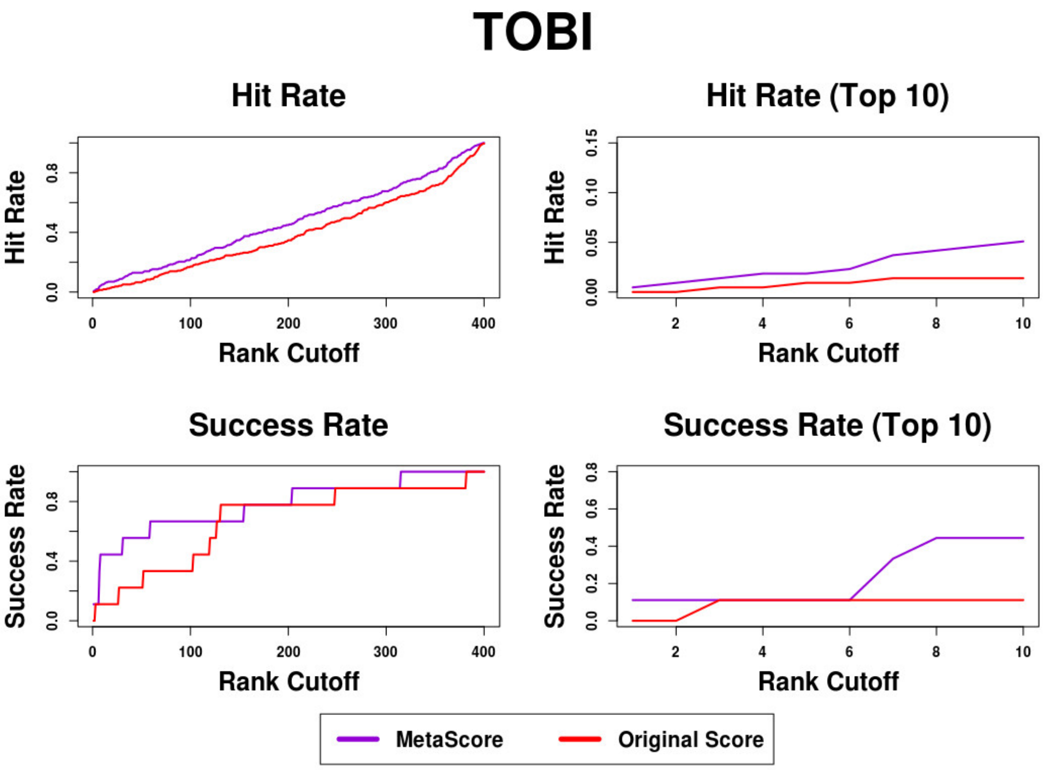

Supplementary Figure S16. Success rates and hit rates plotted against the top m conformations for a classical scoring method (TOBI), machine learning-based method using RF, and the combined method of the two methods using BM5 decoy set. There are four panels. Top-left panel shows hit rates for conformations of top m ranging from 1 to 400; Top-right panel shows hit rates for conformations of top m ranging from 1 to 10; Bottom-left panel shows success rates for conformations of top m ranging from 1 to 400; Bottom-right panel shows success rates for conformations of top m ranging from 1 to 10.

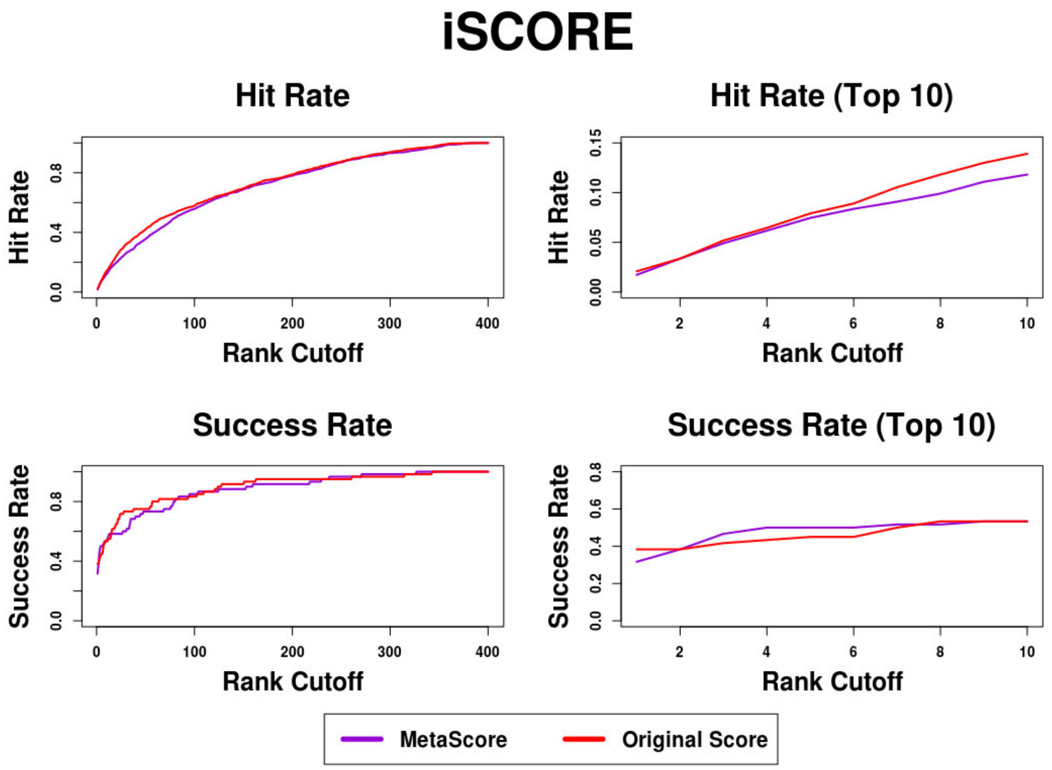

Supplementary Figure S17. Success rates and hit rates plotted against the top m conformations for a classical scoring method (iSCORE), machine learning-based method using RF, and the combined method of the two methods using BM4 decoy set. There are four panels. Top-left panel shows hit rates for conformations of top m ranging from 1 to 400; Top-right panel shows hit rates for conformations of top m ranging from 1 to 10; Bottom-left panel shows success rates for conformations of top m ranging from 1 to 400; Bottom-right panel shows success rates for conformations of top m ranging from 1 to 10.

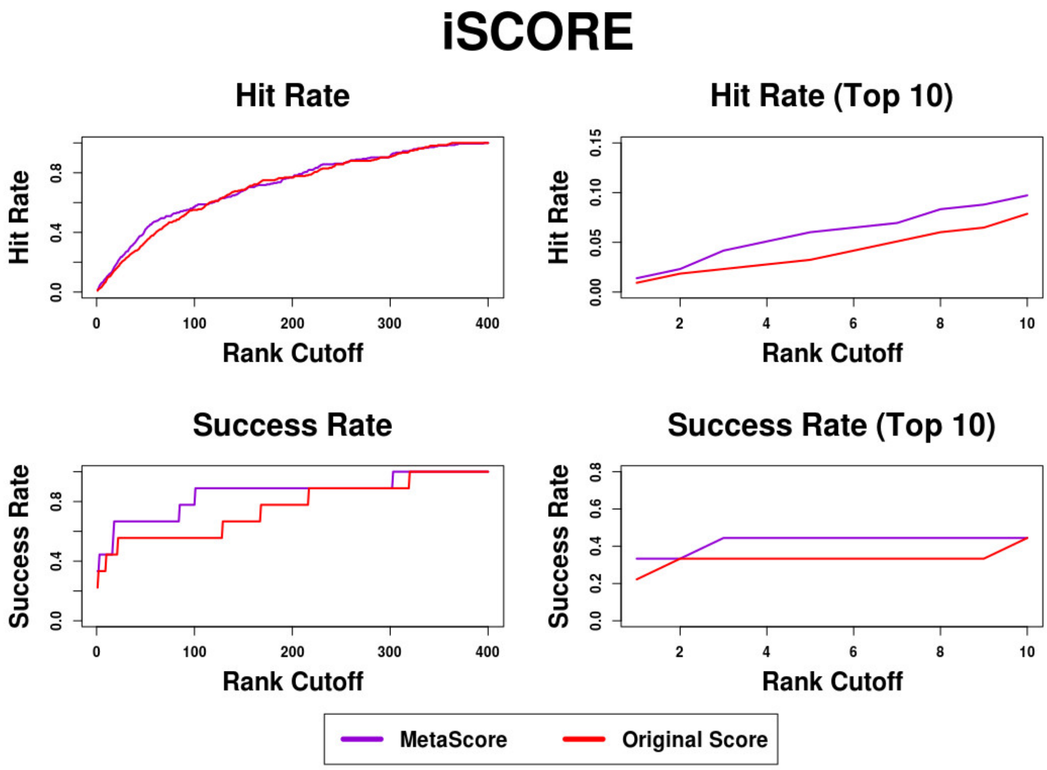

Supplementary Figure S18. Success rates and hit rates plotted against the top m conformations for a classical scoring method (iSCORE), machine learning-based method using RF, and the combined method of the two methods using BM5 decoy set. There are four panels. Top-left panel shows hit rates for conformations of top m ranging from 1 to 400; Top-right panel shows hit rates for conformations of top m ranging from 1 to 10; Bottom-left panel shows success rates for conformations of top m ranging from 1 to 400; Bottom-right panel shows success rates for conformations of top m ranging from 1 to 10.
